# Mortality and treatment costs of hospitalized chronic kidney disease patients between the three major health insurance schemes in Thailand

**DOI:** 10.1186/s12913-016-1792-9

**Published:** 2016-09-29

**Authors:** Sirirat Anutrakulchai, Pisaln Mairiang, Cholatip Pongskul, Kaewjai Thepsuthammarat, Chitranon Chan-on, Bandit Thinkhamrop

**Affiliations:** 1 Department of Medicine, Faculty of Medicine, Khon Kaen University, Khon Kaen Province, 40002 Thailand; 2 Clinical Epidemiology Unit, Faculty of Medicine, Khon Kaen University, Khon Kaen Province, 40002 Thailand; 3 Department of Biostatistics and Demography, Faculty of Public Health, Khon Kaen University, Khon Kaen Province, 40002 Thailand

**Keywords:** Healthcare equity, Healthcare scheme, Chronic kidney disease, Endstage renal disease, Dialysis

## Abstract

**Background:**

Thailand has reformed its healthcare to ensure fairness and universality. Previous reports comparing the fairness among the 3 main healthcare schemes, including the Universal Coverage Scheme (UCS), the Civil Servant Medical Benefit Scheme (CSMBS) and the Social Health Insurance (SHI) have been published. They focused mainly on provision of medication for cancers and human immunodeficiency virus infection. Since chronic kidney disease (CKD) patients have a high rate of hospitalization and high risk of death, they also require special care and need more than access to medicine. We, therefore, performed a 1-year, nationwide, evaluation on the clinical outcomes (i.e., mortality rates and complication rates) and treatment costs for hospitalized CKD patients across the 3 main health insurance schemes.

**Methods:**

All adult in-patient CKD medical expense forms in fiscal 2010 were analyzed. The outcomes focused on were clinical outcomes, access to special care and equipment (especially dialysis), and expenses on CKD patients. Factors influencing mortality rates were evaluated by multiple logistic regression.

**Results:**

There were 128,338 CKD patients, accounting for 236,439 admissions. The CSMBS group was older on average, had the most severe co-morbidities, and had the highest hospital charges, while the UCS group had the highest rate of complications. The mortality rates differed among the 3 insurance schemes; the crude odds ratio (OR) for mortality was highest in the CSMBS scheme. After adjustment for biological, economic, and geographic variables, the UCS group had the highest risk of in-hospital death (OR 1.13;95 % confidence interval (CI) 1.07–1.20; *p* < 0.001) while the SHI group had lowest mortality (OR 0.87; 95 % CI 0.76–0.99; *p* = 0.038). The circumscribed healthcare benefits and limited access to specialists and dialysis care in the UCS may account for less favorable comparison with the CSMBS and SHI groups.

**Conclusions:**

Significant differences are observed in mortality rates among CKD patients from among the 3 main healthcare schemes. Improvements in equity of care might minimize the differences.

**Electronic supplementary material:**

The online version of this article (doi:10.1186/s12913-016-1792-9) contains supplementary material, which is available to authorized users.

## Background

Thailand implemented healthcare reforms in 2002 to ensure universal healthcare provision [1]. The 4 national healthcare insurance schemes include: *(i)* the Universal Coverage Scheme (UCS) provides free medical care for persons without any other insurance (i.e., > 70 % of the population: the majority of farmers, low-income persons, and the unemployed); *(ii)* the Civil Servant Medical Benefit Scheme (CSMBS) provides free medical care for government employees and their dependents; *(iii)* the Social Health Insurance (SHI) scheme for private sector employees; and, *(iv)* private insurance. The first 3 schemes cover > 96 % of the population [2].

The level of healthcare in Thailand depends on the particular hospital type and location. Community hospitals principally provide primary care and have limited resources for treating complex illnesses. Patients from the latter are sent to general (secondary) and tertiary hospitals, as appropriate. The distribution of hospitals in turn depends on economics and geography. The central region—where the capital is located—has the highest gross domestic product (GDP) per capita (~158 % of national GDP). By comparison, the respective proportion of national GDP of the Northeast and North is 34 and 45 % [3].

Previous reports—comparing the fairness of healthcare provision among the 3 main healthcare schemes—were mainly on the provision of medication for cancers and HIV/AIDS [4–8]. To our knowledge, there has been no report comparing the different healthcare schemes vis-à-vis chronic diseases (i.e., chronic kidney diseases – CKD) that require medicine, special care teams, special medical equipment, and hospitalization.

Since CKD patients have an increased rate of hospitalization and a high risk for death [9, 10], we evaluated the nationwide healthcare data of hospitalized CKD patients in fiscal year 2010 for practice outcomes of healthcare among the 3 main health insurance schemes. Our particular focus was on differences in *(i)* clinical outcomes, *(ii)* access to special care and equipment (notably dialysis), and *(iii)* budgeting.

## Methods

The data analyzed were from *(i)* the in-patients total medical expense forms from the UCS fiscal year 2010 from the National Health Security Office; *(ii)* the in-patient data from the CSMBS from the Comptroller General’s Department; and, *(iii)* the in-patient data from the SHI from the Social Security Office. The variables included: sex, age, occupation, address, type of hospital, health insurance scheme, co-morbidities, length of hospital stay (days), complications, treatment, clinical outcomes, and medical expenses (costs charged). Additional information obtained from the Nephrology Society of Thailand, the National Statistical Office, and the Office of the National Economic and Social Development Board, Office of the Prime Minister included: ratio of nephrologists to dialysis units/regional population and end-stage renal disease (ESRD) patients, and the reimbursement of renal replacement therapies among the different healthcare schemes.

The in-patient data were first checked for accuracy by examining for *(i)* overlapping information *(ii)* visit dates *(iii)* missing items *(iv)* incorrect coding and *(v)* the correct fiscal year. CKD patients were identified in either the primary diagnosis (CKD-primary) or secondary diagnosis (CKD-secondary) as code N18 of the International Statistical Classification of Diseases and Related Health Problems, 10th revision (ICD-10) [11]. Hemodialysis and peritoneal dialysis were identified as code 39.95 and 54.98, respectively (ICD-9-CM 2010 classification of procedures) [12]. The data were analyzed not only on the basis of health insurance scheme but also on the level of care provided (i.e., community/primary, general/secondary or tertiary hospital or private hospital) in order to assess accessibility to appropriate care.

### Outcome measures

The differences across the 3 health insurance schemes vis-à-vis in-hospital mortality and high treatment cost were examined. Demographic data, comorbidities and complications were analyzed as to whether they affected the two measures. In addition, policies involving the CKD treatment of the three schemes, budget allocation were explored to facilitate the explanation of the differences of the outcome measures (if any).

### Statistical analysis

STATA version 14 was used for the statistical analyses. The means ± SD or medians (25th-75th percentile) and percentages were used to present the continuous and categorical data, respectively. The generalized estimating equation (GEE) and multiple logistic regression analysis (MLRA) were performed to adjust the odds ratios for factors influencing the *(i)* high cost accounting for multiple admissions within an individual and *(ii)* mortality rate at individual level.

## Results

### Demographic data of the patients

In fiscal 2010, the population over 19 years of age numbered 47,966,734—or 74 % of Thailand’s total population of 64.7 million. Approximately 96 % of the adult population (46,208,964 persons) was covered by one of the 3 health insurance systems. The total number of adult in-patients was 3,876,792 (admitted 4,863,935 times), accounting for 71 % of all in-patients. According to the 23 major disease groups in the ICD 10, among the respective causes of hospitalization and mortality, diseases of the genitourinary system ranked 7th among hospitalized patients (298,258 persons, 7.7 % of all adults in-patients and 392,498 admissions) and the 7th cause of mortality [13].

CKD was the most common diagnosis of the genitourinary system. The total number of CKD patients was 128,338 (generating 236,439 admissions), and accounting for 4.9 % of all adult in-patient admissions (268 persons or 493 visits per 100,000 adult population). Of these, 98,727 persons (185,161 admissions), 24,767 (42,348 admissions) and 4844 (8930 admissions) were covered in the UCS, CSMBS, and SHI groups, respectively.

### Characteristics of hospitalized CKD patients under different healthcare schemes

Table 1 presents the characteristics of CKD patients in the UCS, CSMBS, and SHI schemes. The age of subjects in the CSMBS were the oldest while those in the SHI scheme were the youngest. Most of participants in the UCS, CSMBS and SHI scheme were admitted in a community, tertiary and private hospital, respectively. The patients in the CSMBS and SHI scheme comprised the majority from the central region while those in the UCS were from the Northeast region. Highest proportion of CKD patients in the CSMBS group was diagnosed as CKD-secondary. The respective proportion of ESRD among in-patients under the SHI, CSMBS and UCS was 52.1, 31.6, and 24.2 %.

**Table 1:** Characteristics of CKD patients by the main three health schemes

Characteristics	The main three Thai health schemes		
	Universal Coverage Scheme	Civil Servant Medical Benefit Scheme	Social Health Insurance
Number of adult patients (persons)	98,727	24,767	4844
Number of admissions (times)	185,161	42,348	8930
Age (mean ± SD)	66.55 ± 13.36	72.23 ± 11.46	46.99 ± 12.16
Sex (male/female)	1/1.15	1/0.88	1/0.58
Region (%)			
N/NE/C/S	21.4/46.2/24.2/8.2	17.4/32.6/39.5/10.5	11.6/11.2/71.2/6.1
Hospital levels (%)			
Community hospital	49.95	27.24	1.84
General hospital	23.58	24.49	16.72
Tertiary hospital	22.92	48.14	35.61
Private hospital	3.55	0.13	45.83
Onetime admission/Multiple admission (%)	62.6/37.4	64.8/35.2	60.0/40.0
CKD diagnosed as primary/secondary (%)	62.6/37.424.4/75.6	64.8/35.219.9/80.1	35.1/64.9
Proportion of ESRD ((%)	24.4/75.624.19	19.9/80.131.63	52.06
Common co-morbidities (%)			
Hypertension	56.91	67.90	63.79
Diabetes mellitus	45.08	67.9049.09	36.50
Hyperlipidemia	45.0817.19	49.0925.05	20.05
Ischemic heart disease	13.49	25.0521.68	11.91
Heart failure	13.4914.28	21.6813.95	12.70
Gout	14.2810.49	13.9513.17	6.44
Sepsis	12.78	13.1714.71	10.90
Pneumonia	12.789.94	14.7112.25	8.20
Acute kidney injury	9.948.59	12.2510.26	7.23
Diarrhea	8.598.63	10.268.34	7.51
Stroke	8.636.51	8.3411.41	5.66
Respiratory failure	6.518.53	11.417.85	4.81
Complications (%)			
Anemia requiring blood transfusion	31.86	23.24	31.32
Hyperkalemia	31.8615.95	23.2411.56	11.50
Volume overload	15.9512.44	8.96	14.80
Metabolic acidosis	12.449.20	8.965.04	4.81
Dialysis treatment (% of admissions)			
Hemodialysis	6.97	16.64	24.15
Peritoneal dialysis	6.972.98	16.641.86	2.15
Overall mortality rate (%)	2.9810.39	1.8612.44	8.71
Mortality rate in different hospital levels (%)			
Community/General/Tertiary/Private	3.7/14.9/17.5/13.2	5.9/14.5/15.1/15.2	7.9/8.04/10.4/7.6

Note: *CKD* chronic kidney disease, *ESRD* end stage renal disease, *N* northern region, *NE* northeastern region, *C* central region, *S* southern region, *SD* standard deviation

### Associated co-morbidities

The top 12 diseases associated with CKD patients were hypertension (HT) (59.3 %), diabetes mellitus (DM) (45.5 %), hyperlipidemia (18.8 %), ischemic heart disease (15.0 %), heart failure (14.2 %), sepsis (13.1 %), gout (10.9 %), pneumonia (10.3 %), acute kidney injury on top CKD (8.9 %), diarrhea (8.5 %), respiratory failure (8.3 %) and stroke (7.4 %). Patients in the CSMBS had the greatest proportion of co-morbidities (Table 1).

### Complications

Complications for all CKD patients comprised significant anemia requiring blood transfusion (30.2 %), hyperkalemia (14.9 %), volume overload (11.9 %), and metabolic acidosis (8.2 %). The rate of complications was highest in the UCS (Table 1).

### Dialysis treatment

CKD patients needed dialysis, accounting for 15,684 (12.2 %) patients. The mode of dialysis included hemodialysis (*n* = 12,175; 77.6 %) and peritoneal dialysis (*n* = 3509; 23.4 %). The percentage of those needing hemodialysis was greater under the SHI and CSMBS than the UCS (Table 1). The respective proportion of ESRD patients receiving both types of dialysis during admission under the UCS, SHI and CSMBS was 41.1, 50.5, and 58.5 %.

The characteristics of CKD patients defined as CKD-primary or CKD-secondary and admitted in different hospital levels are presented in the Additional file 1: Table S1 and Additional file 2: Table S2. Subjects in the CKD-secondary group were older, stayed in hospital longer, were more likely from the central region and were admitted to a tertiary hospital. The CKD-secondary group also had more co-morbidities, incurred a higher hospital cost, and had higher mortality rate than the CKD-primary group. In contrast, the rate of complications was higher in the CKD-primary group. Patients treated in tertiary hospitals had more comorbidities; particularly cardiovascular disease, pneumonia, acute on top CKD, and sepsis. By region, hospitals admitting the greatest proportion of CKD patients were in the North and Northeast in community hospitals. By comparison, patients in the central region were admitted to tertiary hospitals. Most of the private hospitals are also located in the central region, where a significant number of patients in the SHI group were admitted.

### Length of hospital stay

The longest hospital stay among CKD patients was in the central region at tertiary hospitals under the CSMBS (Table 2).

**Table 2:** Length of hospital stay and hospital charges for CKD patients by region, hospital level and healthcare scheme

	Length of hospital stays (Days)		Hospital charges (Baht)	
	Mean ± SD	Median (25th-75th percentile)	Mean ± SD	Median (25th-75th percentile)
Insurance				
UCS	5.14 ± 8.01	3.00 (2.00–6.00)	16,040 ± 131,052	6506 (3599–13,562)
CSMBS	9.40 ± 17.81	5.00 (3.00–10.00)	39,401 ± 113,206	12,685 (5694–31,169)
SHI	7.49 ± 11.11	4.00 (3.00–8.00)	36,053 ± 87,854	14,745 (6773–33,588)
Region				
Northern	5.46 ± 7.41	3.00 (2.00–6.00)	15,626 ± 38,605	6796 (3660–14,335)
Northeast	4.56 ± 7.21	3.00 (2.00–5.00)	13,292 ± 164,746	5827 (3378–11,507)
Central	8.50 ± 15.74	5.00 (2.00–9.00)	37,717 ± 114,169	12,549 (5747–30,348)
Southern	6.61 ± 10.91	4.00 (2.00–7.00)	19,443 ± 47,700	7847 (4111–17,058)
Hospital levels				
Community (All schemes)	4.02 ± 4.93	3.00 (2.00–5.00)	7683 ± 144,783	4482 (2830–7548)
- UCS	3.78 ± 4.43	3.00 (1.00–5.00)	7196 ± 154,018	4349 (2775–7196)
- CSMBS	5.85 ± 7.36	4.00 (3.00–7.00)	11,150 ± 24,473	5816 (3387–10,636)
- SHI	6.89 ± 10.35	4.00 (3.00–8.00)	19,013 ± 35,546	8594 (4922–20,042
General (All schemes)	6.56 ± 9.52	4.00 (2.00–7.00)	19,556 ± 119,149	9251 (4967–18,832)
- UCS	5.97 ± 8.77	4.00 (2.00–7.00)	17,969 ± 130,809	8807 (4808–17,531)
- CSMBS	9.08 ± 12.05	6.00 (3.00–10.00)	26,606 ± 56,668	12,179 (6162–25,407)
- SHI	6.85 ± 8.56	4.00 (3.00–8.00)	18,754 ± 45,335	7823 (4152–17,042)
Tertiary (All schemes)	8.64 ± 16.35	5.00 (2.00–9.00)	38,758 ± 102,610	14,404 (6998–32,997)
- UCS	7.21 ± 11.68	4.00 (2.00–8.00)	28,033 ± 65,349	12,043 (6312–25,825)
- CSMBS	11.71 ± 23.47	6.00 (3.00–12.00)	62,964 ± 154,866	22,389 (10,214–54,582)
- SHI	8.65 ± 12.90	5.00 (3.00–9.00)	31,367 ± 71,631	12,724 (5761–28,578)
Private (All schemes)	5.57 ± 8.85	3.00 (2.00–6.00)	43,915 ± 104,269	20,608 (9725–37,617)
- UCS	4.83 ± 7.71	2.00 (1.00–5.00)	42,401 ± 101,864	20,611 (9415–33,539)
- CSMBS	8.46 ± 13.55	5.00 (3.00–7.00)	30,826 ± 71,164	11,732 (7139–24,025)
- SHI	6.86 ± 10.40	4.00 (3.00–7.00)	46,805 ± 108,756	20,820 (10,395–44,565)

Note: *CKD* chronic kidney disease, *UCS* Universal Coverage Scheme, *CSMBS* Civil Servant Medical Benefit Scheme, *SHI* Social Health Insurance, *SD* standard deviation

### Factors influencing the high treatment cost of in-patient

Hospital charges for CKD patients were highest in *(i)* the central region compared with other regions *(ii)* at private hospitals compared with community, general and tertiary hospitals, and *(iii)* covered by CSMBS compared with UCS and SHI (Table 2). Comparing hospital charges of the 3 health schemes with the same hospital levels revealed that hospital charge of the SHI was significantly highest at community hospitals while the CSMBS was the highest at general and tertiary hospitals. No significant differences in hospital charges between the 3 health schemes treated at private hospitals were observed (Table 2).

After adjustment with the factors affecting high hospital charges (>50,000 baht or ~1470 USD per admission)—sex, hospital level, region, co-morbidities, complications, and dialysis treatment—the UCS and SHI groups had a respective 62 and 55 % lower hospital charges than the CSMBS (Table 3).

**Table 3:** Factors influencing high hospital charges (>50,000 baht/1470 USD) among hospitalized, Thai, adult, CKD patients

Variables	No. of admission (times)	No. of high cost admission (%)	Crude odds ratio (95 % CI)	*p*-value	Adjusted odds ratio (95 % CI)	*p*-value
Sex						
Female	126,027	8490 (6.7)	1	<0.001	1	<0.001
Male	110,412	9662 (8.8)	1.31 (1.27–1.36)		1.16 (1.12–1.20)	
Age						
19–30	4376	405 (9.3)	1		1	
31–40	8392	715 (8.5)	0.92 (0.80–1.06)	0.24	1.03 (0.88–1.20)	0.70
41–50	22,172	1682 (7.6)	0.81 (0.72–0.92)	0.001	1.08 (0.94–1.24)	0.26
51–60	46,571	3408 (7.3)	0.77 (0.68–0.86)	<0.001	1.05 (0.92–1.20)	0.49
61–70	62,672	4324 (6.9)	0.72 (0.64–0.81)	<0.001	1.01 (0.89–1.16)	0.85
71–80	64,288	5065 (7.9)	0.82 (0.72–0.92)	0.001	1.02 (0.89–1.16)	0.82
> 80	27,968	2553 (9.1)	0.95 (0.84–1.07)	0.40	1.01 (0.88–1.17)	0.85
Insurance						
CSMBS	42,348	6923 (16.4)	1		1	
SHI	8930	1441 (16.1)	0.99 (0.93–1.06)	0.85	0.45 (0.41–0.49)	<0.001
UCS	185,161	9788 (5.3)	0.29 (0.28–0.30)	<0.001	0.38 (0.36–0.39)	<0.001
Hospital level						
Community	102,251	977 (1.0)	1		1	
General	56,525	4049 (7.2)	7.54 (7.03–8.10)	<0.001	3.58 (3.32–3.87)	<0.001
Tertiary	66,150	10,974 (16.6)	19.36 (18.11–20.70)	<0.001	7.17 (6.67–7.70)	<0.001
Private	11,513	2152 (18.7)	23.09 (21.28–25.05)	<0.001	13.82 (12.58–15.19)	<0.001
Region						
Northern	48,110	2645 (5.5)	1		1	
Northeast	104,067	3703 (3.6)	0.63 (0.60–0.66)	<0.001	0.74 (0.70–0.78)	<0.001
Central	64,962	10,360 (16.0)	3.23 (3.08–3.39)	<0.001	1.83 (1.74–1.94)	<0.001
Southern	19,300	1444 (7.5)	1.36 (1.27–1.46)	<0.001	1.21 (1.12–1.31)	<0.001
Co–morbidities						
Hypertension (yes/no)	125,565/110,874	11,037 (8.8)/7115 (6.4)	1.38 (1.34–1.42)	<0.001	1.00 (0.96–1.04)	0.96
Diabetes mellitus (yes/no)	101,664/134,775	8775 (8.6)/9377 (7.0)	1.27 (1.23–1.31)	<0.001	1.12 (1.08–1.17)	<0.001
Hyperlipidemia (yes/no)	31,229/205,210	3449 (11.0)/14,703 (7.2)	1.51 (1.45–1.57)	<0.001	1.06 (1.01–1.12)	0.016
Ischemic heart disease (yes/no)	29,272/207,167	4406 (15.1)/13,746 (6.6)	2.42 (2.33–2.51)	<0.001	1.75 (1.67–1.84)	<0.001
Heart failure (yes/no)	24,915/211,524	2679 (10.8)/15,473 (7.3)	1.53 (1.46–1.60)	<0.001	1.11 (1.05–1.17)	<0.001
Gout (yes/no)	19,640/216,799	1407 (7.2)/16,745 (7.7)	0.92 (0.87–0.98)	0.005	0.99 (0.93–1.06)	0.76
Sepsis (yes/no)	18,528/217,911	4586 (24.8)/13,566 (6.2)	4.62 (4.44–4.79)	<0.001	2.75 (2.62–2.88)	<0.001
Pneumonia (yes/no)	14,732/221,707	4004 (27.2)/14,148 (6.4)	5.18 (4.98–5.40)	<0.001	3.27 (3.10–3.45)	<0.001
Acute renal failure (yes/no)	12,133/224,306	3351 (27.6)/14,801 (6.6)	5.09 (4.88–5.31)	<0.001	2.35 (2.23–2.48)	<0.001
Diarrhea (yes/no)	12,085/224,354	774 (6.4)/17,378 (7.75)	0.82 (0.76–0.88)	<0.001	0.98 (0.90–1.07)	0.69
Stroke (yes/no)	11,886/224,553	2313 (19.5)/15,839 (7.1)	2.97 (2.83–3.12)	<0.001	1.82 (1.72–1.94)	<0.001
Respiratory failure (yes/no)	11,347/225,092	3080 (27.1)/15,072 (6.7)	5.02 (4.80–5.25)	<0.001	2.30 (2.17–2.44)	<0.001
Complications						
Anemia requiring blood	57,727/178,712	7442 (12.9)/10,710 (6.0)	2.38 (2.31–2.46)	<0.001	2.25 (2.17–2.34)	<0.001
Transfusion (yes/no)						
Hyperkalemia (yes/no)	23,505/212,934	2532 (10.8)/15,620 (7.3)	1.57 (1.51–1.64)	<0.001	1.17 (1.11–1.23)	<0.001
Volume overload (yes/no)	22,091/214,348	1966 (8.9)/16,186 (7.55)	1.30 (1.24–1.36)	<0.001	0.99 (0.93–1.05)	0.67
Metabolic acidosis (yes/no)	11,897/224,542	1549 (13.0)/16,603 (7.4)	1.90 (1.80–2.00)	<0.001	1.11 (1.04–1.19)	0.003
Mode of dialysis						
Hemodialysis	17,143/219,296	5239 (30.6)/12,913 (5.9)	6.45 (6.22–6.70)	<0.001	3.14 (3.00–3.28)	<0.001
Peritoneal dialysis	4584/231,855	1170 (25.5)/16,982 (7.3)	4.16 (3.89–4.46)	<0.001	3.30 (3.04–3.59)	<0.001

Note: *CKD* chronic kidney disease, *UCS* Universal Coverage Scheme, *CSMBS* Civil Servant Medical Benefit Scheme, *SHI* Social Health Insurance, *CI* confidence interval

### Factors associated with mortality

Table 4 presents patient characteristics. After adjustment for age, sex, region, hospital level, hospital charge, co-morbidities, complications, and mode of dialysis, the multiple logistic regression analysis revealed that the highest mortality rate was for patients under the UCS while the lowest was for those under the SHI. Patients under the SHI and CSMBS had a respective 23.0 and 11.5 % reduction of mortality rates compared to the UCS group. Patients who received dialysis had a reduced mortality (hemodialysis; OR 0.90, 95 % CI 0.85–0.96, *p* = 0.002, peritoneal dialysis; OR 0.87, 95 % CI 0.78–0.96, *p* = 0.006). Other factors influencing the mortality rate included *(i)* elderly age *(ii)* level of care (i.e., tertiary hospitals had higher mortality rates than general and private hospitals while community hospital had lowest rate); *(iii)* presence of ESRD; *(iv)* co-morbidities (viz., sepsis, respiratory failure, stroke, pneumonia, acute on top CKD, ischemic heart disease, heart failure, and DM); and *(v)* complications of CKD (i.e., metabolic acidosis, hyperkalemia and volume overload) (Table 5).

**Table 4:** Characteristics of dead and alive hospitalized CKD patients

Characteristics	Discharge status of CKD patients		
	Dead CKD patients	Alive CKD patients	*p*-value
Number of patients (persons)	13,755	114,583	
Age (years; mean ± SD)	67.89 ± 14.04	66.79 ± 13.70	<0.001
Sex (male/female)	1/0.98	1/1.07	<0.001
Health scheme (%)			
UCS/CSMBS/SHI	74.5/22.4/3.1	77.2/18.9/3.9	<0.001
Hospital levels (%)			
Community/General/Tertiary/Private			
First admission	24.6/29.7/41.0/4.7	46.1/22.7/26.7/4.5	<0.001
Frequent admission	17.3/33.0/44.9/4.8	42.7/24.2/28.5/4.5	<0.001
Last admission	14.9/34.6/45.9/4.6	43.4/24.2/28.0/4.4	<0.001
Onetime admission/Multiple admission (%)	54.6/45.4	63.9/36.1	<0.001
CKD diagnosed as primary/secondary (%)	21.7/78.3	24.2/75.8	<0.001
Proportion of ESRD ((%)	39.29	25.16	<0.001
Common co-morbidities (%)			
Hypertension	59.54	59.26	0.52
Diabetes mellitus	49.40	45.06	<0.001
Hyperlipidemia	17.43	18.98	<0.001
Ischemic heart disease	23.10	14.04	<0.001
Heart failure	23.82	13.00	<0.001
Gout	10.12	10.94	0.003
Sepsis	44.68	9.29	<0.001
Pneumonia	28.06	8.19	<0.001
Acute kidney injury	21.61	7.33	<0.001
Diarrhea	8.30	8.56	0.31
Stroke	15.06	6.51	<0.001
Respiratory failure	34.22	5.14	<0.001
Complications (%)			
Anemia requiring blood transfusion	43.01	28.64	<0.001
Hyperkalemia	27.10	13.47	<0.001
Volume overload	20.58	10.81	<0.001
Metabolic acidosis	20.35	6.77	<0.001
Dialysis treatment (%)			
Hemodialysis	18.29	8.43	<0.001
Peritoneal dialysis	5.21	2.44	<0.001
Length of stay (days; mean ± SD)	11.4 ± 24.8	5.8 ± 8.8	<0.001
Hospital charges (baht; mean ± SD)	61,662 ± 164,842	18,656 ± 55,546	<0.001

Note: *ESRD* end stage renal disease, *CKD* chronic kidney disease, *ESRD* end stage renal disease, *UCS* Universal Coverage Scheme, *CSMBS*Civil Servant Medical Benefit Scheme, *SHI* Social Health Insurance, *SD* standard deviation

**Table 5:** Prognostic factors influencing mortality rates among hospitalized, Thai, adult, CKD patients

Variables	No. of patients (persons)	Dead persons and mortality rate (%)	Crude odds ratio (95 % CI)	*p*-value	Adjusted odds ratio (95 % CI)	*p*-value
Sex						
Female	66,134	6814 (10.3)	1		1	
Male	62,204	6941 (11.2)	1.09 (1.06–1.13)	<0.001	1.03 (0.99–1.07)	0.17
Age						
19–30	1869	189 (10.1)	1		1	
31–40	4135	400 (9.7)	0.95 (0.79–1.14)	0.60	1.16 (0.95–1.42)	0.15
41–50	10,556	1127 (10.7)	1.06 (0.90–1.25)	0.47	1.28 (1.07–1.54)	0.008
51–60	23,073	2318 (10.0)	0.99 (0.85–1.16)	0.93	1.21 (1.01–1.44)	0.039
61–70	32,837	3277 (10.0)	0.99 (0.84–1.15)	0.85	1.25 (1.05–1.50)	0.013
71–80	37,727	4039 (10.7)	1.07 (0.91–1.24)	0.42	1.40 (1.18–1.68)	<0.001
> 80	18,141	2405 (13.3)	1.36 (1.16–1.59)	<0.001	1.82 (1.52–2.19)	<0.001
Insurance						
CSMBS	24,767	3080 (12.4)	1		1	
UCS	98,727	10,253 (10.4)	0.82 (0.78–0.85)	<0.001	1.13 (1.07–1.20)	<0.001
SHI	4844	422 (8.7)	0.67 (0.60–0.75)	<0.001	0.87 (0.76–0.99)	0.038
Hospital level						
Community	56,151	3384 (6.0)	1		1	
General	30,156	4091 (13.6)	2.45 (2.33–2.57)	<0.001	1.58 (1.50–1.68)	<0.001
Tertiary	36,274	5634 (15.5)	2.87 (2.74–3.00)	<0.001	1.62 (1.53–1.71)	<0.001
Private	5757	646 (11.2)	1.97 (1.80–2.15)	<0.001	1.51 (1.35–1.68)	<0.001
Hospital charges (Baht/USD)						
First quartile (<5550/< 163)	32,090	701 (2.2)	1		1	
Second quartile (5550–13,271/163–390)	32,079	2028 (6.3)	3.02 (2.77–3.30)	<0.001	2.08 (1.90–2.28)	<0.001
Third quartile (13,272–34,502/390–1015)	32,085	3680 (11.5)	5.80 (5.34–6.30)	<0.001	2.98 (2.72–3.26)	<0.001
Fourth quartile (>34,502/> 1015)	32,084	7346 (22.9)	13.30 (12.28–14.39)	<0.001	4.43 (4.03–4.88)	<0.001
Onetime admission/Multiple admission	80,764/47,574	7514 (9.3)/6241 (13.1)	1.47 (1.42–1.53)	<0.001	0.64 (0.61–0.68)	<0.001
CKD diagnosed as primary/secondary	30,731/97,607	2991 (9.7)/10,764 (11.0)	1.15 (1.10–1.20)	<0.001	0.95 (0.90–1.01)	0.11
ESRD (yes/no)	34,234/94,104	5405 (15.8)/8350 (8.9)	1.93 (1.86–2.00)	<0.001	1.49 (1.42–1.57)	<0.001
Co-morbidities						
Diabetes mellitus (yes/no)	58,427/69,911	6795 (11.6)/6960 (10.0)	1.19 (1.15–1.23)	<0.001	1.12 (1.08–1.17)	<0.001
Ischemic heart disease (yes/no)	19,264/109,074	3178 (16.5)/10,577 (9.7)	1.84 (1.76–1.92)	<0.001	1.43 (1.35–1.51)	<0.001
Heart failure (yes/no)	18,174/110,164	3277 (18.0)/10,478 (9.5)	2.09 (2.01–2.18)	<0.001	1.38 (1.31–1.46)	<0.001
Sepsis (yes/no)	16,792/111,546	6146 (36.6)/7609 (6.8)	7.89 (7.58–8.20)	<0.001	4.28 (4.09–4.48)	<0.001
Pneumonia (yes/no)	13,247/115,091	3860 (29.1)/9895 (8.6)	4.37 (4.19–4.56)	<0.001	1.59 (1.51–1.68)	<0.001
Acute renal failure (yes/no)	11,367/116,971	2972 (26.1)/10,783 (9.2)	3.49 (3.33–3.65)	<0.001	1.44 (1.36–1.52)	<0.001
Stroke (yes/no)	9527/118,811	2071 (21.7)/11,684 (9.8)	2.55 (2.42–2.68)	<0.001	1.85 (1.73–1.96)	<0.001
Respiratory failure (yes/no)	10,596/117,742	4707 (44.4)/9048 (7.7)	9.60 (9.19–10.03)	<0.001	3.64 (3.45–3.83)	<0.001
Complications						
Anemia requiring blood Transfusion (yes/no)	38,730/89,608	5916 (15.3)/7839 (8.7)	1.88 (1.81–1.95)	<0.001	1.03 (0.98–1.07)	0.28
Hyperkalemia (yes/no)	19,165/109,173	3728 (19.5)/10,027 (9.2)	2.39 (2.29–2.49)	<0.001	1.48 (1.40–1.55)	<0.001
Volume overload (yes/no)	15,213/113,125	2,831 (18.6)/10,924 (9.7)	2.14 (2.04–2.24)	<0.001	1.23 (1.16–1.31)	<0.001
Metabolic acidosis (yes/no)	10,561/117,777	2,799 (26.5)/10,956 (9.3)	3.52 (3.35–3.69)	<0.001	1.72 (1.62–1.82)	<0.001
Mode of dialysis						
Hemodialysis (yes/no)	12,175/116,163	2,516 (20.7)/11,239 (9.7)	2.43 (2.32–2.55)	<0.001	0.90 (0.85–0.96)	0.002
Peritoneal dialysis (yes/no)	3,509/124,829	717 (20.4)/13,038 (10.4)	2.20 (2.02–2.39)	<0.001	0.87 (0.78–0.96)	0.006

Note: *CKD* chronic kidney disease, *ESRD* end stage renal disease, *UCS* Universal Coverage Scheme, *CSMBS* Civil Servant Medical Benefit Scheme, *SHI* Social Health Insurance, *CI* confidence interval

In addition to the factors associated with mortality, health policies among the health schemes differed (Table 6). Patients in the UCS trended to have fewer benefits than patients in the other healthcare schemes. Patients under the UCS were not able to choose the hospitals with full-scale CKD care. They had to be referred by a primary care hospital. The limited distribution of nephrologists and dialysis units outside major urban centres might be a barrier for patients under the UCS who live mainly in the North and Northeast (Table 7).

**Table 6:** Comparison the health policies on the health care providing and reimbursement among the three schemes

Issues	SHI	UCS	CSMBS
Financial barriers to equitable access	Acute complications occurred, the patients had access to special care without barrier by referral system.		
Administrative efficiency	Three health care schemes applied the same clinical practice guidelines		
Patient and provider autonomy	The patients received the care from only the self-registry hospitals (either private or public hospitals). Treatment of ESRD was hemodialysis or CAPD depended on facility of the self-registry hospitals.	The patients received care only the public hospitals in their casement areas, mostly community or general hospitals. The patients were referred to higher facility hospital whenever the complications occurred. CAPD was the first treatment for ESRD patients.	The patients freely chosen the public or tertiary care hospital that they preferred. Physicians had autonomy to choose hemodialysis or CAPD for treatment of ESRD.
Non-financial barriers to equitable access	Most of the patients worked in the big cities in Bangkok and central region of Thailand which had better population/nephrologist ratio than UCS	Most of the patients were in the rural area that had least population/nephrologist ratio	Most of the patient chosen to be care in the tertiary care hospitals and medical school hospitals which had best population/nephrologist ratio
Reimbursement of erythropoietin administration			
- Pre-dialysis	No	No	Yes
- Dialysis	Yes	Yes	Yes
Reimbursement of dialysis for ESRD patients			
- CAPD	Yes	Yes	Yes
- Hemodyalysis	Not more than 1,500 bahts (44 USD)/session and not more than 4,500 bahts (132USD)/week.	Hemodialysis was allowed only CAPD was contraindication or having complications. Not more than 1,500–1,700 bahts (44–50 USD)/session and not more than 3,000–3,400 bahts (88–100 USD)/week.	As the actual expenses

Note: *ESRD* end stage renal disease, *CAPD* continuous ambulatory peritoneal dialysis, *SHI* Social Health Insurance, *UCS* Universal Coverage Scheme, *CSMBS* Civil Servant Medical Benefit Scheme

**Table 7:** Comparing the ratio of population and ESRD patients per one nephrologists and one dialysis unit in different regions

Regional health care	Population/nephrologist (n)	Population/dialysis unit (n)	ESRD patients/nephrologist (n)	ESRD patients/dialysis unit (n)
Bangkok	44,177	51,585	71	83
Central part	204,307	152,801	99	74
Northern	529,820	233,121	221	97
Northeastern	592,690	197,563	229	76
Southern	422,429	173,941	144	59

Note: *ESRD* end stage renal disease

## Discussion

CKD is defined as abnormalities in the kidney structure or function and/or a decreased glomerular filtration rate for more than 3 months (GFR < 60 ml/min/1.73 m^2^) [14]. Code N18 in the ICD-10 represents an older nomenclature for chronic renal failure as a decrease in GFR comparable to stage 3a-5 CKD patients (estimated GFR < 60 ml/min/1.73 m^2^). Our study revealed that 45–60 % of admitted CKD patients also had hypertension and diabetes. The co-morbidities associated with mortality and high hospital charges were sepsis, pneumonia, respiratory failure followed by cardiovascular diseases and AKI on pre-existing CKD. This finding agrees with previous studies that demonstrated the severity of CKD increased in-hospital mortality among patients with acute coronary syndrome [15–20], heart failure [21–23], cardiac surgery [24], and stroke [25]. Early optimum therapeutic interventions and appropriate medications might improve clinical outcomes and reduce the cost of hospitalization.

After the Thai healthcare reforms were implemented in 2002, the poor indeed had wider access to medical services. Equity of health financing, health workers and healthcare infrastructure have been studied and an improving trend in equity was reported [26–29]. Notwithstanding, differences in access to hospital types persist among the 3 insurance schemes. The population under UCS must be registered at a community hospital near home. When necessary, there is a line of referrals. Any patient who does not follow the referral process and attempts to go directly to a tertiary care center will have to pay all costs by themselves. By comparison, under the CSMBS, a government employee can register at any public hospital according to their preference [2] while an employee covered by SHI must register at the contracted public or private hospital [30]. If a referral is required, the employee must go to one of the hospitals in the designated network. Only in an emergency may persons covered by SHI or UCS be exempted from paying; however, they must be transferred to their registered hospital as soon as possible. These vagaries in regulations provide an explanation as to why those on UCS go to community hospitals and government employees go to tertiary care hospitals [2]. Since the UCS group comprises a higher proportion of low socio-economic patients, mainly located in rural region, they experience delayed hospital accessibility. Furthermore, our study revealed differences in clinical outcomes of hospitalized CKD patients that might represent residual inequality that needs addressing.

Mortality rates of hospitalized CKD patients differed among the 3 insurance schemes: the crude odd ratios revealed the highest mortality under the CSMBS. Patients admitted under the CSMBS had more severe or complicated disease than the other schemes; as indicated by the highest *(i)* percentage of life-threatening co-morbidities, *(ii)* length of stay, and *(iii)* hospital charges. After adjusting for biological and economic geographic variables, the multivariable analysis demonstrated that the UCS group had the highest risk of in-hospital death while the SHI group had the lowest mortality. The explanation may be related to the limited health care benefits under the UCS compared to the CSMBS and SHI. Table 6 presents a comparison of the health policies among the 3 schemes. The CSMBS appears to have better benefits than the other schemes; such as free access to specialist care, free service without capitation, and better chances of getting kidney replacement therapy (either hemodialysis or CAPD) for ESRD patients. Furthermore, the ratios of population and ESRD patients per nephrologist and dialysis unit were the best in Bangkok and the central region: that is, the regions where a higher proportion of patients are under either the CSMBS or SHI. On the other hand, in the other regions where most patients are under the UCS, poorer ratios prevail. The insufficiency of medical personnel and equipment might be the reasons for higher CKD complications and less dialysis treatment in the UCS group. Previous studies confirmed that remote CKD patients were less likely to receive specialist care, to receive laboratory testing, and to get appropriate medications, and were more likely to die or be hospitalized compared with those living closer to a nephrologist [31, 32].

Improving CKD care might be achieved by implementing policies that ensure fairness by providing a comparable budget allocation among the healthcare schemes. The “PD First” policy in Thailand launched in 2008 initiated CAPD as renal replacement therapy for ESRD patients under the UCS [33]. This policy represents an effective strategy for correcting the inadequate distribution of hemodialysis machines and insufficient numbers of nephrologists in rural areas and to underprivileged groups.

Anemia is one of the complications seen in CKD patients, and this can be corrected by injection of erythropoiesis stimulating agent (ESA). ESA is relatively costly and is only reimbursed during the pre-dialysis period under the CSMBS scheme. Our data revealed that there was a lower proportion of patients with anemia requiring blood transfusion under the CSMBS than the UCS or SHI groups. More intensive, high-cost medication support by the 3 main health schemes might reduce morbidity and hospitalization.

The strength of this study is that almost all of the subjects were hospitalized adult, Thai, CKD patients. The results, therefore, provide a clear overview of the situation vis-à-vis these adult patients; however, some limitations existed. Lack of a registered nationwide laboratory system means that there is no standardized staging of CKD patients, which might influence the clinical outcomes. The present study analyzed the administrative claim data, therefore socio-economic status of patients was not available. In addition, we are not able to generate an area locator as a proxy for socio-economic status of individual patients due to lack of data. Insufficient data of these demand-side characteristics observed at the individual level made some limitations in comparison of equity among the three health insurance schemes. The record of charges for each group represents an average and this might not wholly characterize the severity of individual patients nor include details of the procedures and medical instruments needed for each patient. Moreover, the mortality focused in this study was outcome at discharge which may be different with mortality after discharge because some patients died at home.

## Conclusions

Concerning the treatment of hospitalized CKD patients, the UCS group had the poorest healthcare benefits compared to the other healthcare schemes—e.g., less budget for hospital care (charge cost), poorer access to specialist care, and treatment options that depended on variable healthcare policies. These might explain the greater mortality rate of those under the UCS compared to those under the CSMBS or SHI. In order to improve health outcomes among hospitalized CKD patients, new healthcare policies are needed to improve budget allocations, accessibility to specialist care, and distribution of resources.

## Additional files

Additional file 1: Table S1. Presented characteristics of CKD patients by primary, secondary and total (combined primary and secondary) diagnoses. (DOCX 26 kb) Additional file 2: Table S2. Focused on characteristics of CKD patients by the hospital levels. (DOCX 28 kb)

## Abbreviations

AKI: Acute kidney injury
CAPD: Continuous ambulatory peritoneal dialysis
CI: Confidence interval
CKD: Chronic kidney disease
CSMBS: The Civil Servant Medical Benefit Scheme
DM: Diabetes mellitus
ESA: Erythropoiesis stimulating agent
ESRD: End stage renal disease
GDP: Gross domestic product
GEE: Generalized estimating equation
GFR: Glomerular filtration rate
HIV/AIDS: Human immunodeficiency virus infection and acquired immune deficiency syndrome
HT: Hypertension
ICD: International Statistical Classification of Diseases and Related Health Problems
MLRA: Multiple logistic regression analysis
OR: Odds ratio
SD: Standard deviation
SHI: The Social Health Insurance
UCS: The Universal Coverage Scheme
