# Erratum to: Mortality and treatment costs of hospitalized chronic kidney disease patients between the three major health insurance schemes in Thailand

**DOI:** 10.1186/s12913-016-1874-8

**Published:** 2016-10-25

**Authors:** Sirirat Anutrakulchai, Pisaln Mairiang, Cholatip Pongskul, Kaewjai Thepsuthammarat, Chitranon Chan-on, Bandit Thinkhamrop

**Affiliations:** 1 Department of Medicine, Faculty of Medicine, Khon Kaen University, Khon Kaen, Khon Kaen Province 40002 Thailand; 2 Clinical Epidemiology Unit, Faculty of Medicine, Khon Kaen University, Khon Kaen, Khon Kaen Province 40002 Thailand; 3 Department of Biostatistics and Demography, Faculty of Public Health, Khon Kaen University, Khon Kaen, Khon Kaen Province 40002 Thailand

## Erratum

Upon publication of this article [1], it was brought to our attention that Table 1 contained several errors. The correct Table 1 is shown below:

**Table 1:** Characteristics of CKD patients by the main three health schemes

Characteristics	The main three Thai health schemes		
	Universal Coverage Scheme	Civil Servant Medical Benefit Scheme	Social Health Insurance
Number of adult patients (persons)	98,727	24,767	4,844
Number of admissions (times)	185,161	42,348	8,930
Age (mean ± SD)	66.55 ± 13.36	72.23 ± 11.46	46.99 ± 12.16
Sex (male/female)	1/1.15	1/0.88	1/0.58
Region (%)			
N / NE / C / S	21.4 / 46.2 / 24.2 / 8.2	17.4 / 32.6 / 39.5 /10.5	11.6 / 11.2 / 71.2 / 6.1
Hospital levels (%)			
Community hospital	49.95	27.24	1.84
General hospital	23.58	24.49	16.72
Tertiary hospital	22.92	48.14	35.61
Private hospital	3.55	0.13	45.83
Onetime admission / Multiple admission (%)	62.6 / 37.4	64.8 / 35.2	60.0 / 40.0
CKD diagnosed as primary / secondary (%)	24.4 / 75.6	19.9 / 80.1	35.1 / 64.9
Proportion of ESRD (%)	24.19	31.63	52.06
Common co-morbidities (%)			
Hypertension	56.91	67.90	63.79
Diabetes mellitus	45.08	49.09	36.50
Hyperlipidemia	17.19	25.05	20.05
Ischemic heart disease	13.49	21.68	11.91
Heart failure	14.28	13.95	12.70
Gout	10.49	13.17	6.44
Sepsis	12.78	14.71	10.90
Pneumonia	9.94	12.25	8.20
Acute kidney injury	8.59	10.26	7.23
Diarrhea	8.63	8.34	7.51
Stroke	6.51	11.41	5.66
Respiratory failure	8.53	7.85	4.81
Complications (%)			
Anemia requiring blood transfusion	31.86	23.24	31.32
Hyperkalemia	15.95	11.56	11.50
Volume overload	12.44	8.96	14.80
Metabolic acidosis	9.20	5.04	4.81
Dialysis treatment (% of admissions)			
Hemodialysis	6.97	16.64	24.15
Peritoneal dialysis	2.98	1.86	2.15
Overall mortality rate (%)	10.39	12.44	8.71
Mortality rate in different hospital levels (%)			
Community / General / Tertiary / Private	3.7 / 14.9 / 17.5 / 13.2	5.9 / 14.5 / 15.1 / 15.2	7.9 / 8.04 / 10.4 / 7.6

*CKD* chronic kidney disease, *ESRD* end stage renal disease, *N* northern region, *NE* northeastern region, *C* central region, *S* southern region, *SD* standard deviation
