# Supplementary material for: Mortality and treatment costs of hospitalized chronic kidney disease patients between the three major health insurance schemes in Thailand
Source: BMC Health Serv Res. 2016 Sep 29;16:528. doi: 10.1186/s12913-016-1792-9 (PMC5043539; doi:10.1186/s12913-016-1792-9)
Supplement: Additional file 1: Table S1. — Presented characteristics of CKD patients by primary, secondary and total (combined primary and secondary) diagnoses. (DOCX 26 kb) [file 12913_2016_1792_MOESM1_ESM.docx]

**Table S1: Characteristics of CKD patients by primary, secondary and total (combined primary** **and secondary) diagnoses**

| **Characteristics** | **CKD patients defined as** | | |
| --- | --- | --- | --- |
|  | **Primary diagnosis** | **Secondary diagnosis** | **Total (Combined primary and secondary diagnoses)** |
| **Number of adult patients (persons)**  **Number of admissions (times)**  **Age (mean ± SD)**  **Sex (male/female)**  **Hospital levels (%)**  Community / General / Tertiary / Private  **Region (%)**  N / NE / C / S  **Health schemes (%)**  UCS/CSMBS/SHI  **Onetime admission / Multiple admission (%)**  **Proportion of ESRD ((%)**  **Common co-morbidities (%)**  Hypertension  Diabetes mellitus  Hyperlipidemia  Ischemic heart disease  Heart failure  Gout  Sepsis  Pneumonia  Acute kidney injury  Diarrhea  Stroke  Respiratory failure  **Complications (%)**  Anemia requiring blood transfusion  Hyperkalemia  Volume overload  Metabolic acidosis  **Dialysis treatment (%)**  Hemodialysis  Peritoneal dialysis  **Length of stay (days)**  Mean ± SD  Median (25^th^-75^th^ percentile)  **Hospital charge (baht)**  Mean ± SD  median (25^th^-75^th^ percentile)  **Mortality rate (%)** | 30,731  66,274  64.08 ± 14.67  1/1.27  48.9 / 22.3 / 22.9 / 5.9  22.2 / 45.4 / 24.3 / 8.1  78.44 / 16.02 / 5.54  58.3 / 41.7  55.39  62.89  40.84  15.92  9.35  11.19  10.18  8.70  6.87  4.98  6.81  3.94  8.11  47.50  21.71  26.40  11.88  17.40  6.17  5.14 ± 9.03  3.00 (2.00 - 5.67)  16,762 ± 63,228  7,160 (4,028 - 14,579)  9.73 | 97,607  170,165  67.80 ± 13.31  1/1.01  42.1 / 23.9 / 30.0 / 4.0  19.7 / 41.3 / 30.4 / 8.6  76.45 / 20.33 / 3.22  30.4 / 69.6  17.63  58.15  47.00  19.72  16.79  15.10  11.07  14.47  11.41  10.08  9.07  8.52  8.30  24.72  12.80  7.27  7.08  7.00  1.65  6.79 ± 12.45  4.00 (2.00 -7.00)  25,313 ± 80,047  9,044 (4,645 - 20,442)  11.03 | 128,338  236,439  66.91 ± 13.74  1/1.06  43.7 / 23.5 / 28.3 / 4.5  20.3 / 42.3 / 28.9 / 8.5  76.93 / 19.30 / 3.77  37.1 / 62.9  26.67  59.29  45.53  18.81  15.01  14.16  10.86  13.08  10.32  8.86  8.53  7.42  8.26  30.18  14.93  11.85  8.23  9.49  2.73  6.40 ± 11.74  4.00 (2.00 - 7.00)  23,265 ± 76,445  8,516 (4,469 - 18,909)  10.72 |

Note: ESRD; end stage renal disease, N; northern region, NE; northeastern region, C; central region, S; southern region, UCS**;** Universal Coverage Scheme,

CSMBS; Civil servant medical benefit scheme, SHI; Social Health Insurance, SD; standard deviation
