# Supplementary material for: Mortality and treatment costs of hospitalized chronic kidney disease patients between the three major health insurance schemes in Thailand
Source: BMC Health Serv Res. 2016 Sep 29;16:528. doi: 10.1186/s12913-016-1792-9 (PMC5043539; doi:10.1186/s12913-016-1792-9)
Supplement: Additional file 2: Table S2. — Focused on characteristics of CKD patients by the hospital levels. (DOCX 28 kb) [file 12913_2016_1792_MOESM2_ESM.docx]

**Table S2**: Characteristics of CKD patients by the hospital levels

| **Characteristics** | **The hospital levels** | | |  |
| --- | --- | --- | --- | --- |
|  | **Community hospital** | **General hospital** | **Tertiary hospital** | **Private hospital** |
| **Number of adult patients (persons)**  **Number of admissions (times)**  **Age (mean ± SD)**  **Sex (male/female)**  **Health schemes (%)**  UCS / CSMBS / SHI  **Region (%)**  N / NE / C / S  **Onetime admission / Multiple admission (%)**  **CKD diagnosed as primary / secondary (%)**  **Proportion of ESRD ((%)**  **Common co-morbidities (%)**  Hypertension  Diabetes mellitus  Hyperlipidemia  Ischemic heart disease  Heart failure  Gout  Sepsis  Pneumonia  Acute kidney injury  Diarrhea  Stroke  Respiratory failure  **Complications (%)**  Anemia requiring blood transfusion  Hyperkalemia  Volume overload  Metabolic acidosis  **Dialysis treatment (% )**  Hemodialysis  Peritoneal dialysis  **Overall mortality rate (%)**  **Mortality rate in different health schemes**  UCS / CSMBS / SHI | 56,151  102,251  68.54 ± 12.55  1/1.23    87.8 / 12.0 / 0.2  21.5 / 56.3 / 14.7 / 7.5  60.2 / 39.8  26.8 / 73.2  20.31    57.41  46.05  16.80  9.99  12.41  11.73  9.91  8.57  5.28  10.62  4.35  5.48  26.21  14.48  12.16  7.99    3.82  1.63  6.03  6.0 / 6.1 / 9.0 | 30,156  56,525  66.24 ± 13.89  1/1.02  77.2 / 20.1 / 2.7  24.1 / 37.5 / 27.2 / 11.2  66.0 / 34.0  22.8 / 77.2  27.86  55.74  43.46  16.88  16.47  15.65  10.32  15.66  10.82  9.01  7.76  8.26  11.28  35.50  15.58  11.64  9.47  10.28  3.51  13.57  13.6 / 14.0 / 8.0 | 36,274  66,150  66.32 ± 14.46  1/0.92  62.4 / 32.9 / 4.7  17.3 / 28.6 / 44.9 / 9.2  65.6 / 34.4  19.4 / 80.6  34.35  64.26  46.50  23.04  20.87  15.24  10.50  16.27  12.82  14.45  6.08  11.45  10.63  31.73  15.60  11.16  7.30    16.63  4.03  15.53  16.1 / 15.2 / 10.3 | 5,757  11,513  58.13 ± 15.40  1/0.81    60.9 / 0.6 / 38.5  7.3 / 16.1 / 76.5 / 0.1  57.0 / 43.0  31.5 / 68.5  34.22    64.88 45.11  21.94  19.37  16.71  7.38  10.46 9.05 7.73  7.64  7.59 4.50    31.21  11.83 14.38  9.94  15.56  1.25  11.22  13.4 / 12.1 / 7.7 |

Note: ESRD; end stage renal disease, UCS; Universal Coverage Scheme, CSMBS; Civil Servant Medical Benefit Scheme**,** SHI; Social Health

Insurance, N; northern region, NE; northeastern region, C; central region, S; southern region**,** SD; standard deviation
